# Considerably Unfolded Transthyretin Monomers Preceed and Exchange with Dynamically Structured Amyloid Protofibrils

**DOI:** 10.1038/srep11443

**Published:** 2015-06-25

**Authors:** Minna Groenning, Raul I. Campos, Daniel Hirschberg, Per Hammarström, Bente Vestergaard

**Affiliations:** 1 Department of Pharmacy and Department of Drug Design and Pharmacology, University of Copenhagen, Copenhagen, Denmark; 2 IFM – Department of Chemistry, Linköping University, Linköping, Sweden

## Abstract

Despite numerous studies, a detailed description of the transthyretin (TTR) self-assembly mechanism and fibril structure in TTR amyloidoses remains unresolved. Here, using a combination of primarily small -angle X-ray scattering (SAXS) and hydrogen exchange mass spectrometry (HXMS) analysis, we describe an unexpectedly dynamic TTR protofibril structure which exchanges protomers with highly unfolded monomers in solution. The protofibrils only grow to an approximate final size of 2,900 kDa and a length of 70 nm and a comparative HXMS analysis of native and aggregated samples revealed a much higher average solvent exposure of TTR upon fibrillation. With SAXS, we reveal the continuous presence of a considerably unfolded TTR monomer throughout the fibrillation process, and show that a considerable fraction of the fibrillating protein remains in solution even at a late maturation state. Together, these data reveal that the fibrillar state interchanges with the solution state. Accordingly, we suggest that TTR fibrillation proceeds via addition of considerably unfolded monomers, and the continuous presence of amyloidogenic structures near the protofibril surface offers a plausible explanation for secondary nucleation. We argue that the presence of such dynamic structural equilibria must impact future therapeutic development strategies.

Transthyretin (TTR) is a 55 kDa homotetrameric β-sheet-rich protein mainly produced in the liver, being responsible for the transport of the hormone thyroxine and vitamin A^1^,^2^. TTR amyloid diseases are associated with four protein aggregation ‘gain of toxic function diseases’, which are senile systemic amyloidosis (SSA), familial amyloidotic polyneuropathy (FAP), familial amyloidotic cardiomyopathy (FAC), and familial leptomeningeal amyloidosis. TTR associated diseases are frequently occurring, SSA is e.g. acquired by carriers of wild type (WT) TTR^3^,^4^ and affects up to 25% of the population over 80 years old^4^. The process of amyloidosis is linked to tissue degeneration, yet amyloid fibrils themselves may not mediate the cytotoxicity. In fact several studies support a cytotoxic role for lower molecular mass soluble species formed during TTR amyloidogenesis^5^,^6^,^7^,^8^. Only limited treatments are available for the TTR amyloid diseases such a liver transplantation for the FAP patients^9^ or a newly approved medication for treatment of FAP^10^. It is hence of utmost importance to gain insight into the molecular processes underlying TTR amyloidosis in order to stimulate further therapeutic development.

Various models are proposed for the self-assembly mechanism of TTR into fibrils. These mechanistic models can roughly be divided into two groups. One group considers the fibril formation to occur by monomer addition^11^,^12^,^13^,^14^,^15^,^16^. The other group suggests the fibrils to be formed by oligomeric building blocks (dimers or a multimer of such) and is thereby questioning whether dissociation of dimers into monomers is a prerequisite for amyloid formation^17^,^18^,^19^. For the first group of models the fibrillation mechanism of TTR has been proposed to be a downhill polymerization process^11^,^12^,^13^, where the tetramer dissociation rather than nucleation of fibril growth is the rate-limiting step^14^. The pathway of TTR tetramer dissociation is suggested by Kelly and co-workers to occur by scission of the tetramer along the crystallographic C_2_ axis in the reported crystal structure (e.g. 1TTA.pdb^20^) affording the formation of dimers that rapidly dissociate into monomers^21^. The alternative oligomeric assembly mechanism is supported by the ability of some stabilized TTR dimers to fibrillate, e.g. fibrillation is possible even when the two protomers in the dimers are chemically cross-linked^22^. In addition a tetramer (a dimer of dimers) has also been proposed to be the building block of TTR amyloid fibrils, based on molecular packing observed in the crystal structure of a highly amyloidogenic variant of TTR^23^. Generally, both types of models agree that a partial unfolding of the building block is essential for fibril formation^14^,^24^,^25^,^26^,^27^ and that the process may proceed through fibrillogenic intermediate states, termed protofibrils. In this work a protofibril is defined according to Kodali and Wetzel as a relatively short flexible curvilinear aggregate which has an on- or off-pathway role in fibril formation^28^. The amyloid fibrils are defined as relatively straight, unbranched protein aggregates with diameters in the 10 nm range, that often consist of multiple protofilaments twisted around the fibril axis^28^. Various fibrillogenic structures have been observed for TTR including amyloid fibrils^29^,^30^,^31^ and curvilinear protofibrils^8^,^32^,^33^. Clearly the curvilinear protofibrils often observed *in vitro* are not similar to the *ex vivo* amyloid fibrils but may represent *in vivo* fibril precursors. Another possibility is that the protofibrils are off-pathway, as suggested by an extended study where, under certain conditions, transition from WT TTR protofibril bundles to mature fibrils did not occur even after one year^33^.

Recently small-angle X-ray scattering (SAXS) studies have been applied to the fibrillation process of insulin, glucagon and α-synuclein showing the presence, kinetics and low-resolution structures of accumulating oligomers on the pathway of fibril formation^34^,^35^,^36^. SAXS is very appropriate for such studies since the structural changes of fibrillating proteins are followed in solution without any labelling or surface effects. Moreover, since the scattering contribution from multiple existing species can be deconvoluted^37^,^38^, the process can be followed without disturbing the equilibrium of the evolving fibrillation. Here, we supplement the SAXS analysis with Thioflavin T (ThT) fluorescence, transmission electron microscopy (TEM), Fourier transform infrared spectroscopy (FTIR), circular dichroism (CD) and in particular hydrogen exchange mass spectrometry (HXMS). Our analysis not only results in structural evidence, yielding a model for the mechanism of TTR protofibril formation, it also reveals an unexpectedly dynamic interchange between a considerably unfolded solution state of TTR and the protofibril state. Our report hence uncovers structural principles, which are fundamentally different from previously characterized systems. The presence of such an amyloidogenic monomer can explain fundamental properties of fibrillation kinetics. We suggest that continued focus on the considerably unfolded TTR monomer, which is highlighted for the first time in this study, is of key importance for further advancement in drug development against TTR amyloidoses.

## Results

### Formation of curvilinear protofibrils

**Figure 1:**
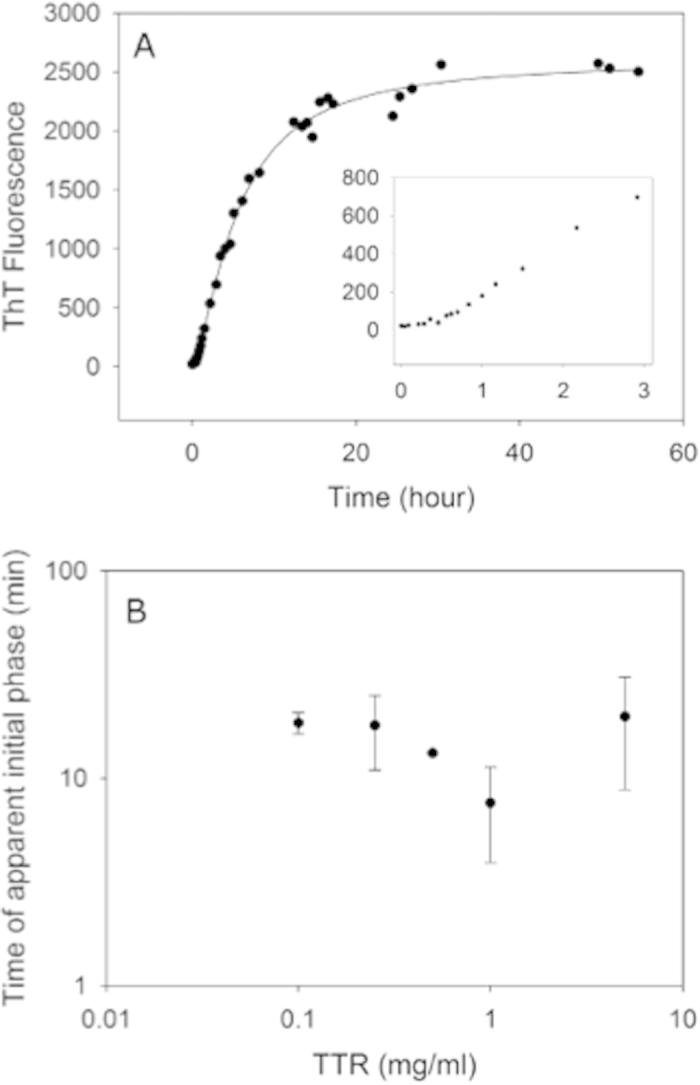
Transthyretin fibril formation measured by Thioflavin T fluorescence. (**A**) Fibril formation of 5 mg/ml transthyretin (TTR) in 50 mM acetic acid with 100 mM NaCl (pH 3.0) at 4 °C measured by *ex situ* Thioflavin T fluorescence at pH 7.5. The solid line is meant as a guide for the eye. The insert is a zoom on the first three hours of fibrillation. The apparent initial lag-phase is estimated to 20 ± 11 minutes. (**B**) The time of the initial lag-phase of TTR fibrillation is independent of the protein concentration within the examined concentration range.

**Figure 2:**
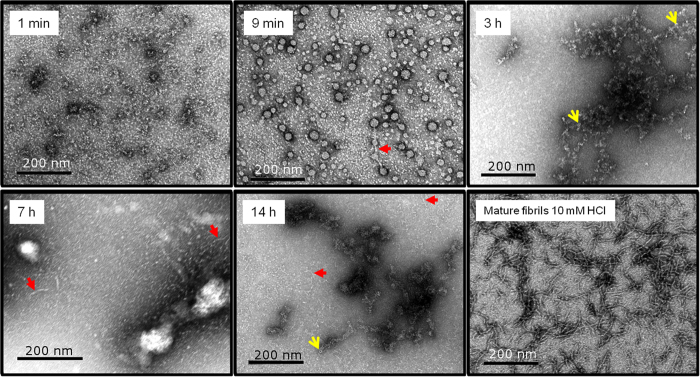
Transmission electron microscopy (TEM) micrographs of the transthyretin fibrillation process. Already after 9 minutes small curvilinear protofibrils are revealed, apparently protruding between some spherical aggregates. These fibrillar structures, having a diameter of 5–10 nm are the most dominant species present throughout the fibrillation process. Mature fibrils formed in 10 mM HCl and 10 mM NaCl at 22 °C for >one month are also shown. The red filled arrows point at elongated protofibrils and the yellow arrow heads on annules, i.e. circular protofibrils. The scale bars represent 200 nm.

Prior to fibrillation, TTR was kept in demineralised water (dH_2_O), and fibrillation was initiated by the addition of acetic acid and NaCl. As revealed by *ex situ* ThT fluorescence analysis (Fig. 1A), only a slow increase in fluorescence signifying fibril formation was observed during the first twenty minute short lag-phase, after changing the experimental conditions to fibril-promoting conditions. TEM micrographs reveal that already during the brief apparent lag-phase (one minute after initiation), small ill-defined aggregates were visualised (Fig. 2) and after 9 minutes of incubation, a few small protofibrils protruding between spherical aggregates were further visualised, hence corresponding to the very subtle increase in ThT fluorescence observed. The spherical aggregates were heterogenic in size with diameters in the range from 15 to 40 nm. The apparent lag-phase was followed by a rapidly evolving second growth phase revealed by increasing ThT fluorescence values. This growth phase lasted for approximately 20 hours of incubation, after which only limited progress was detected (Fig. 1A).

TEM images during the second growth phase (3, 7, and 14 hours of incubation) revealed mainly TTR curvilinear protofibrils having a diameter of approximately 5–10 nm and a length of around 25–75 nm (Fig. 2). In addition a small proportion of annular protofibrils were observed among the curvilinear protofibrils. No μm-scale amyloid-like fibrils were observed in the micrographs. Varying the TTR concentration in the range 0.1 to 5 mg/ml did not change the length of the lag-phase (Fig. 1B). For comparison, long (up to μm) mature protofibrils visualized after one month in 10 mM HCl, 100 mM NaCl, 22 °C had a comparable diameter of around 5-10 nm and no annular structures were observed.

### SAXS analysis of the initial state and early fibrillation time-points reveals the presence of a considerably unfolded TTR monomer

**Figure 3:**
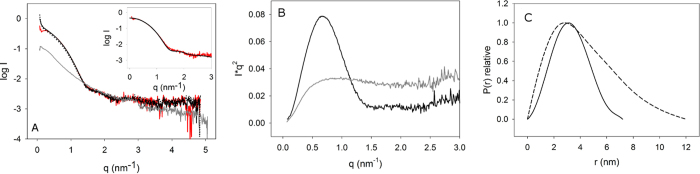
SAXS scattering curves, Kratky representation and relative pair distance distribution functions. **(A)** Scattering curves, revealing intensity on a logarithmic scale, against the scattering vector q (q = 4π sin θ/λ, 2θ equals the scattering angle). Transthyretin (TTR) prior to fibrillation (red) is superposed with scattering curves recorded 3½ minutes (black) and 10 minutes (dotted) after initiation. Furthermore, the measured scattering curve of an unfolded TTR monomer is shown in grey. The inset shows the scattering curve of TTR prior to fibrillation (red) fitted to the calculated curve from the tetrameric crystal structure (using the crystallographic coordinates 1TTA.pdb). **(B)** Kratky representation (I(q)*q^2^ versus q) for tetrameric TTR prior to initiation (black) and the considerably unfolded TTR monomer in 11% acetic acid (grey). It is evident that TTR prior to initiation primarily consists of folded protein while the Kratky representation of TTR in 11% acetic acid corresponds to a Gaussian coil. **(C)** Relative pair distance distribution function (P(r)) of tetrameric TTR in dH_2_O with a D_max_ of 7.2 nm (black) and TTR in 11% acetic acid with a D_max_ of 12 nm (dashed). The P(r) functions are scaled to a maximum value of 1.

**Figure 4:**
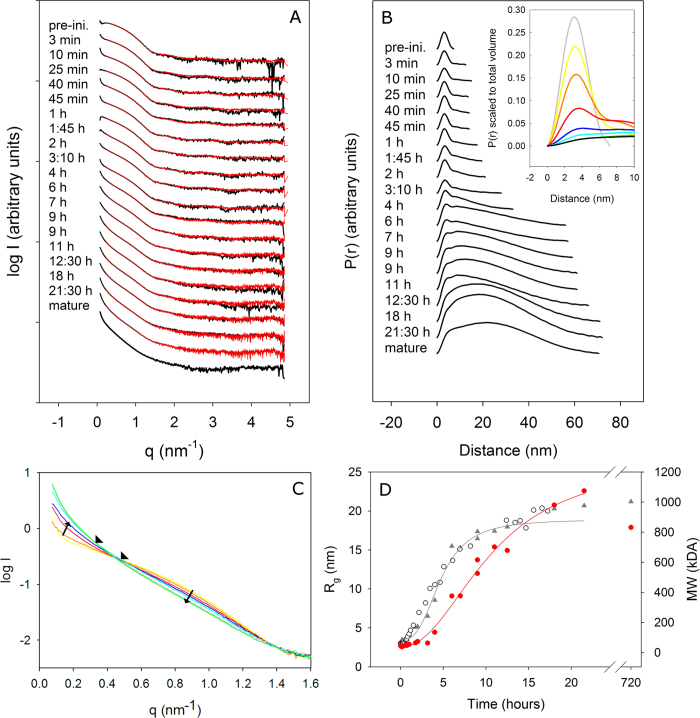
Transthyretin fibrillation process described using SAXS data. **(A)** SAXS scattering intensity against the scattering vector q (q = 4π sin θ/λ, 2θ equals the scattering angle) at different time points during the fibrillation (black) and the fitted curves (red). The fitted curves are obtained by linear combination of the scattering curves for the pre-initiation tetramer and protofibril after 21½ hours of incubation. Curves show pre-initiation condition (top), kinetics (3½ min to 21½ hours) and mature protofibrils (1 month) (bottom). Curves are translated arbitrarily to provide a clear view of each curve. **(B)** Normalized pair distance distribution function (P(r)) calculated from the corresponding scattering curves. The sequence of curves follows that of the raw scattering curves in A and again curves are translated arbitrarily for viewing purposes. The inset shows selected P(r) functions scaled to the same volume. Pre-initiation (grey), 40 minutes (yellow), 2 hours (orange), 4 hours (red), 7 hours (blue), 11 hours (cyan), 21½ hours (green), and mature protofibrils (black). **(C)** Zoom of the lowest scattering angles of SAXS scattering curves at 40 minutes (yellow), 2 hours (orange), 4 hours (red), 7 hours (blue), 11 hours (cyan), 18 hours (grey), and 21½ hours (green) after fibrillation initiation. Arrows indicate the time development and cross-over points are marked with arrow heads. **(D)** The estimated average radius of gyration (R_g_) and average molecular weight (MW) at various time points during the fibrillation process. R_g_ is estimated using GNOM (grey triangles). MW estimates (red spheres) are based on the scattering intensity at zero angle (I_0_) from the Guinier approximation. The solid lines are guides for the eye. For comparison the ThT fluorescence intensity (open spheres) are normalised on a scale from three to 20. In all panels the data from the outlier at 14½ hours of incubation are left (included in Table S1).

We collected SAXS data from TTR in the pre-fibrillation conditions (in dH_2_O), during the fibrillation process, and in 11% acetic acid. It is immediately evident from the raw scattering curves and the Kratky representation (Figs 3A,B) that TTR in 11% acetic acid is in a considerably unfolded form. This is evident from the rather featureless scattering curve and the monotonous increase until reaching a plateau in the Kratky representation^39^. It is further evident from the extrapolated forward scattering (I_0_) that the molecular weight under these conditions corresponds to the monomeric form (Supporting Table S1). As revealed by the pair distance distribution functions (P(r)) (Figs 3C and 4B), pre-initiation and early-fibrillation states are predominantly globular, while the featureless P(r) function in 11% acetic acid also is typical for a predominantly unfolded state, revealing a dramatically increased maximal dimension (D_max_). The main difference between pre-initiation and early fibrillation conditions can be ascribed to the presence of a small amount of early large aggregates (Fig. 3A), in accordance with the ThT fluorescence and TEM analysis (Figs 1A and 2). It is evident that neither the pre-initiation state nor the early time-points correspond to the native tetrameric structure. Firstly, from the Guinier approximation an average radius of gyration (R_g_) of 2.6 nm was estimated in the pre-initiation state and the calculated mean molecular weight (MW) is 45 kDa (~3.3 monomers on average or 15% monomer and 85% tetramer) (Supporting Table S1). Accordingly, the theoretical scattering curve of the tetrameric crystal structure (1TTA.pdb) yielded a poor overall fit to the scattering curve of TTR in dH_2_O (Fig. 3A inset) particularly evident at intermediate scattering angles. The fit was also not improved when altering the tetramer structure, by applying rigid body refinement (data not shown). Hence, together with the clear indication from the lowered average MW, it was excluded that minor structural rearrangements could explain the divergence between the solution structure and the crystal structure.

As the MW estimate suggests presence of species smaller than tetramers in the pre-initiation solution (Supporting Table S1), it was investigated whether the scattering could be interpreted as a mixture of tetrameric and smaller-molecular weight oligomers of the native species (i.e. mixtures of monomers, dimers and tetramers). For this, the theoretical scattering curves for tetrameric, dimeric and monomeric native structures were calculated (isolated from either the native crystal structure 1TTA.pdb, or the “native-like” crystal structure 3CBR.pdb). It was, however, not possible to obtain improved fits to the experimental curves by a linear combination of these scattering curves. This clearly indicates the presence of a non-native species in solution. Hence, we attempted to fit a linear combination of the calculated scattering curve from tetrameric native TTR and the experimental scattering curve for non-native monomeric TTR in 11% acetic acid. This resulted in greatly improved fits when using 15% non-native monomer and 85% tetramer, in accordance with the estimated MW (Supporting Table S2).

### SAXS study in solution reveals the overall TTR fibrillation process

SAXS measurements were performed during fibrillation, by extracting individual samples from the fibrillating solution (Fig. 4A). At the lower scattering angles significantly increasing intensities and steeper slopes reveal that the species grow dramatically in size while changing their overall shape from a spherical to an elongated appearance. P(r) functions reveal the average distribution of pair distances for all scatterers present in solution at each time point (Fig. 4B). Pronounced bell-shaped features with a peak maximum around 3.1 nm, similar to evident features in the TTR pre-initiation scattering curve, remain visible for several hours of fibrillation. Already at early time points (3–10 minutes), D_max_ and the general proportion of larger distances increase significantly hence confirming that longer shapes are present already at early stages (Supporting Table S1 and Fig. 4B). The final approximate D_max_ of 70 nm is in accordance with those observed by TEM. It is noteworthy that the D_max_ values at 18 h and 21:30 h are larger than the final D_max_ after 1 month of maturation. In this context, it should be noted that a second cross-over point of the superposed scattering curves appears at late time-points (see Fig. 4C). This cross-over point appears only after the ThT signal is steady, and thus signifies a maturation of the fibril state, rather than newly formed aggregates. The P(r) functions reveal clearly elongated features, but also still exhibit significant features from the tetramer. The latter is evidenced by the peak/shoulder maximum around 3.1 nm.

After a slow initial phase of around 45 minutes the progress of R_g_ (Fig. 4D) reveals a steep growth phase for about 10 hours, followed by a significantly slower growth phase up to the latest measurement around 21½ hours. This development is in complete agreement with the ThT fluorescence development (Fig. 4D). Only a slight further increase in R_g_ is observed in the very mature sample (720 hours). The general Guinier approximation of R_g_ is compromised due to the presence of very elongated species in solution even at early time points, but these values are included for comparison (Supporting Table S1). The development of the average MW is initially slower than the progress of R_g_ and has a steeper increase at later time points (Fig. 4D). This suggests that significant unfolding (leading to larger R_g_) preceeds oligomerisation (leading to larger MW). The estimated R_g_ and MW of the mature protofibril samples are comparable to samples after 18 and 21½ hours. There is hence no indication of further association into thicker or longer fibrils upon long-term incubation.

**Figure 5:**
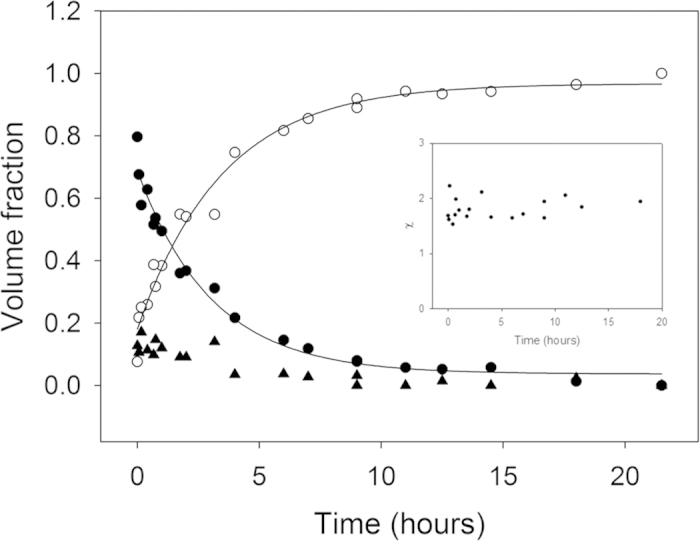
Deconvolution of scattering curves. The calculated volume fractions are shown for the measured scattering curve for the unfolded monomer (solid triangles), the theoretical scattering curve for the crystallographic (1TTA) tetramer (solid spheres) and the measured scattering curve for the protofibrils after 21½ hours of incubation (open spheres). To guide the eye the volume fractions of tetramer are fitted by a single exponential decay function (3 parameters) (solid spheres) and a single exponential rise to maximum (3 parameters) for the fibril (open spheres). The discrepancy between the fit of the deconvoluted scattering curves and the experimental data as a function of time is plotted in the inset.

As mentioned above, when superposing the scattering curves throughout the fibrillation process, two crossover points were observed (Fig. 4C). This suggests the presence of at least three main species, because the total scattering is a linear combination of the scattering contribution from each of the individual species present in solution. Two of the species were present during the entire process, while a new species appeared after 10 hours (when the second crossover point appears). Accordingly, a singular value decomposition (SVD) of the entire data set suggests that three components contribute significantly to the scattering (Supporting Figure S1), including two main and one minor dominating species. Based on the observation of the late second crossover point, we suggest that these correspond to a pre-fibrillar state, an early fibril state, and a late fibrillar state. To further elaborate on the above suggestion, we attempted to fit the measured scattering curves with scattering from individual components. We employed the program OLIGOMER, which fits experimental curves with linear combinations of input scattering curves, the latter being either theoretical or experimental curves. Scattering curves measured at all late fibrillar states were tested in combination with curves from early states (data not shown) but most combinations were not able to adequately explain the progression in the SAXS data. However, good fits were obtained throughout the fibrillation process (Fig. 5) when combining the scattering curve measured after 21½ hours with the experimental scattering curve, measured for unfolded TTR in acetic acid and a scattering curve calculated from the crystallographic tetramer (1TTA.pdb). It can be seen from Fig. 5 that the proportion of considerably unfolded monomer is a nearly constant volume fraction of the entire pool of scatterers. This means that the proportion between folded states and the unfolded state is constant. It is also possible to obtain adequate fits to the experimental data, by combining the scattering curve measured after 21½ hours with the scattering curve measured for the pre-initiation state (supplementary Figure S2). This is in accordance with our previous conclusion, that the pre-initiation state consists of tetramer and unfolded monomer.

When using the scattering curve from the matured protofibril sample, much poorer fits were obtained throughout the fibrillation process, suggesting that the final protofibril state is not an adequate representation of the protofibrils that are present at earlier timepoints. This is again in agreement with the late crossover points observed. This means that the conversion to the final protofibril state happens at much slower time rates than the formation of the initial protofibrils. It is hence evident that a final maturation process takes place, after the initial elongation process. Fitting to the data set was also attempted using calculated scattering curves representing native monomers, dimers and modified tetramers in combination with the fibril scattering curves, all such attempts yielding very poor fits (data not shown).

Our analysis leads to three major conclusions: Firstly, we showed the presence of a constant ratio of considerably unfolded monomers and structured states composed of native tetramers and protofibrils throughout the fibrillation process. Secondly, no accumulation of any new oligomeric species was revealed. Neither native monomers nor dimers were detected. Thirdly, it was evident that both early and more mature protofibrillar species were formed (Fig. 4C). Our analysis clearly shows that the early fibril is formed during the elongation phase at a time-rate much higher than the mature fibril visualized after one month (720 h) of maturation. This mature protofibril has a shorter global D_max_ than the protofibrils after 21½ hours. We can thus conclude that the maturation leads to a gradual homogenisation of the length of protofibrils.

### Structural analysis of the TTR mature protofibrils by SAXS and HXMS

**Figure 6:**
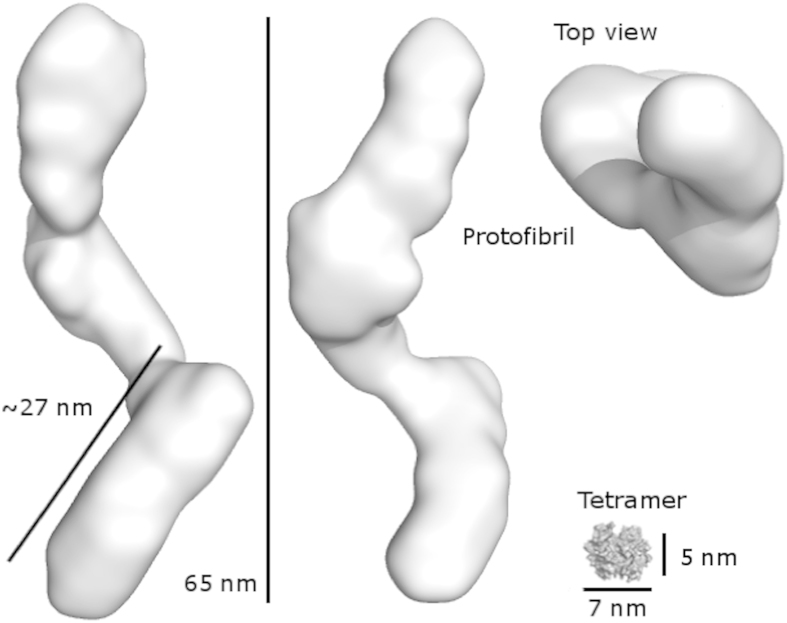
*Ab initio* modelling of a mature TTR protofibril. The filtered average model of the protofibril is shown in three views where the model is rotated 90° around its long axis and 90° to show the top view. The model has a length of 65 nm and a diameter of 13 nm. The surface representation of the TTR tetramer (1TTA.pdb) is shown at scale with the fibril. The approximate dimension of the native tetrameric TTR is 7 × 5 × 4 nm. All images are visualised using PyMol. The PyMOL Molecular Graphics System, Version 1.5.0.4 Schrödinger, LLC.

The above analysis indicates that the 21½ hours sample includes both early and late protofibrils, while the sample of one month (720 h) is more homogeneous. A SAXS solution structure of the mature TTR protofibrils was hence constructed from this sample. 20 independent *ab initio* reconstructions of the protofibril were made (Supporting Figure S3), revealing a general elongated and twisted appearance. In accordance with the curvilinear and heterogeneous appearance evident from TEM images, the *ab initio* models are significantly different, having an average normalized spatial discrepancy (NSD) value of 1.43 ± 0.05^40^. However, at a lower resolution, the models had many common overall features. The program DAMAVER provided an average of the aligned individual models and a filtered compact model visualising the most typical protofibril structure. This structure is an elongated and twisted fibril structure, with a length of around 65 nm (Fig. 6), whereas the average length of individual models was 72 ± 1 nm. The hydration volume of the protofibril was 4.94 × 10^6 ^Å^3^, corresponding to an approximate molecular weight of about 2900 kDa or 215 TTR monomers.

**Figure 7:**
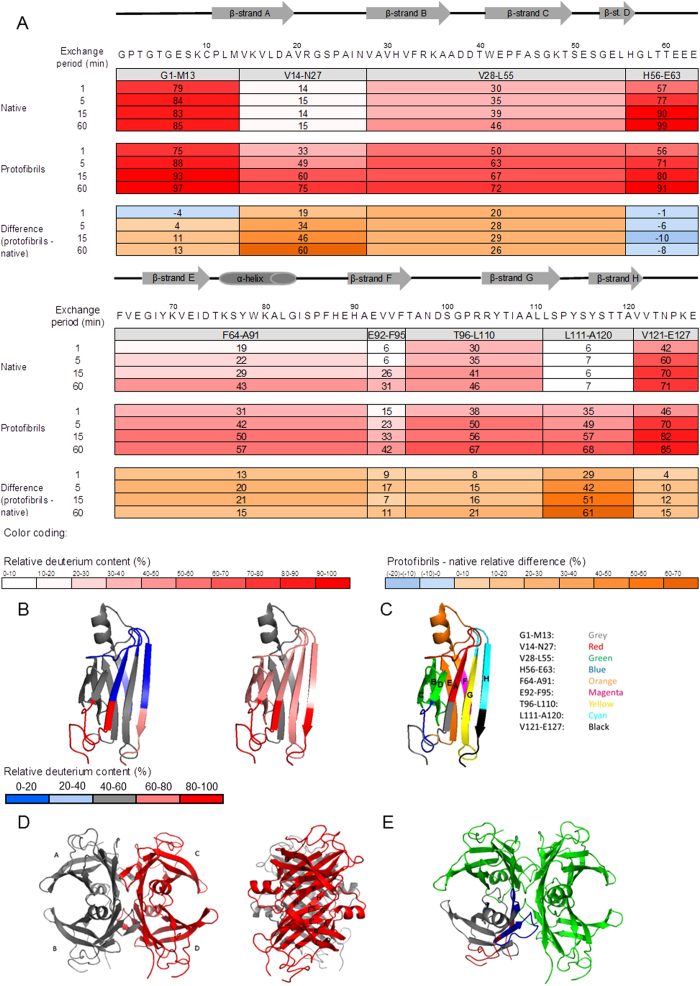
Hydrogen exchange by mass spectrometry (HXMS) performed at pH 3 and room temperature. **(A)** Heat map representation of relative deuterium contents (in percent) for native and mature (3 weeks old) TTR protofibrils. Peptic peptides are represented by gray boxes, and their relative deuterium content is indicated below as a function of exchange time. Differential heat maps are shown below, and are based on the absolute percentage point difference in relative deuterium content between protofibrillar and native TTR. The secondary structure components of native TTR are shown above the primary sequence. **(B)** Heat map of relative deuterium content after 1 hour of exchange time for native and protofibrillar TTR, projected on the 1TTA.pdb crystal structure. **(C)** Peptic peptides map projected on the 1TTA.pdb crystal structure, where the β-strands have been labelled A-H. **(D)** TTR tetramer folded as a dimer of dimers shown in two views, related by a 90° rotation. The AB dimer (grey) and the CD dimer (red) are shown. Each monomer contains eight β-strands located within the 127 amino acid protein as follows (marked on two protomers on the right hand view): A (11-18), B(27-37), C(39-50), D(53-55), E(67-73), F(91-97), G(103-112) and H(115-123). Each dimer contains two β-sheets: CBEFF’E’B’C’(outer sheet) and DAGHH’G’A’D’(inner sheet). A short α-helix is present from residues 75 to 82. The approximate dimensions of one protomer are 3.5 × 2.5 × 4 nm. The tetrameric TTR crystal structure (1TTA.pdb^20^) is visualized using PyMol (The PyMol Molecular Graphics System, Version 1.5.0.4 Schödinger, LLC). **(E)** The monomer in figure **(B)** revealing the heat map of relative deuterium content for the native tetrameric species, is shown in context of the tetramer (green).

For a further investigation of the structure of TTR protomers in the protofibril structure, we applied HXMS analysis. HXMS allows assessing the solvent protection of protein backbone amides via a measure of exchange of hydrogens. Protection is known to arise at least in part from persistent hydrogen bonded structure^16^, and decreased protection is often interpreted as an increased structural flexibility. The native TTR and mature protofibrils were analysed to determine segment-specific solvent protection. The experimental conditions trigger aggregation for the initially native TTR sample. Therefore, we mainly focused on the first hour of the reaction, since until then minimal formation of aggregates is detected by ThT fluorescence (Fig. 1A inset). The two samples showed a significantly different protection pattern for several segments. Overall, solvent protection was much lower for protofibrils compared to native protein (Fig. 7). The three most protected segments in the native tetramer were V14-N27, E92-F95 and L111-A120 (Fig. 7B,E). The V14-N27 peptic peptide comprises part of the A strand and the whole loop connecting A and B strands. The latter is a hydrophobic dimer:dimer interaction region (Fig. 7E). The E92-F95 segment is part of the F strand, which is involved in F-F’ monomer:monomer interactions. L111-A120 comprises the H strand and the loop connecting the G and H strands, involved in the monomer:monomer H-H’ and hydrophobic dimer:dimer interfaces, respectively (Fig. 7E). The three least protected segments of native TTR were G1-M13, H56-E63 and V121-E127. The first and the third of these segments belong to the N- and C-terminal regions, respectively. These data are hence fully in accordance with the presence of a tetramer during pre-initiation conditions. More explicitly, the association of two monomers into a dimer forms a β-sandwich stabilised by hydrogen bonds between the H-H’ and F-F’ strands. The dimer is composed of two eight-stranded β-sheets, DAGHH’G’A’D’ and CBEFF’E’B’C’. This dimer binds through hydrophobic interactions between the A-B and G-H loops to another dimer (Fig. 7D). Thus, our HXMS results on native TTR are well in accordance with the TTR crystal structure (PDB ID 1TTA).

HXMS performed on the protofibrils showed that the most protected segment was E92-F95, situated on the F strand (Fig. 7). The least protected segments were G1-M13, H56-E63 and V121-E127, similar to the protection measured for native TTR. The major differences between the protofibril and the native tetramer were in segments V14-N27 and L111-A120, which become less protected towards HXMS upon fibrillation. This corresponds with the TTR fibril structure model at pH 4.4 generated by Serag *et al.*^26^, where the F strand is part of the core and the H strand is exposed to solvent. However, we could not reproduce Serag’s data on the protected 29–33 segment. The regions generally involved in the formation of both dimers and tetramers are significantly less protected in the protofibrils than in native TTR (Fig. 7B,E). This hence indicates that the dimer and tetramer interfaces have been disrupted upon fibrillation and, rather surprisingly, that the average protomer in the protofibril sample is in a conformation which is not consistent with a persistent hydrogen-bonded structure.

### Secondary structure of mature TTR protofibrils studied using FTIR and CD

**Figure 8:**
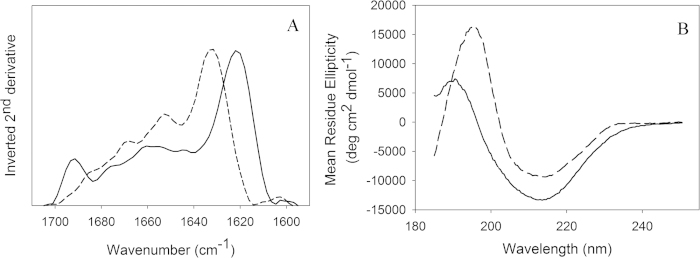
Secondary structure determined by FTIR and CD. **(A)** Fourier transform infrared spectroscopy of 4.5 mg/ml TTR in 50 mM phosphate buffer with 100 mM NaCl (pH 7.5) (dashed line) and TTR protofibrils formed of 4.5 mg/ml WT TTR in 50 mM acetic acid with 100 mM NaCl (pH 3.0) incubated at 4 °C for about 1 month (solid line). The inverted 2^nd^ derivative spectra in the Amide I region are shown. (**B**) Far UV circular dichroism of native TTR (dashed line) and TTR protofibrils (solid line). The distribution of the structural element fractions are shown in Table S3. CD accordingly revealed a considerably rearranged structure of protofibrils compared to native TTR (Fig. 8B), as also observed using different fibrillation protocols^24^,^51^,^80^. The TTR crystal structure contains 5% α-helix, 48% β-sheet and 47% turns and disordered components. This is in good agreement with the experimentally observed 6% α-helix, 45% β-sheet and 49% remainder secondary structure components deconvoluted from the CD spectra (Supporting Table S3). Protofibrils, judging from the deconvoluted CD spectra, contain 13% α-helix, 33% β-sheet and 54% of turns and disordered secondary structure. Thus, an increased percentage of turns and disordered regions are observed for protofibrils. However, the suggested increase in α-helical content may be an artifact due to the lack of a reference database for proteins having a highly fibrous and “disordered” structure^81^, as previously observed^82^.

The highly dynamic nature of the protein backbone of mature protofibrils shown by HXMS implied significant loss of secondary structure compared to native tetrameric TTR as expected from the long incubation in denaturing conditions. FTIR spectra were recorded of H_2_O solutions of native TTR and mature TTR protofibrils (Fig. 8A). The native β-sheet-rich TTR has an amide I maximum around 1632 cm^−1^ associated with the presence of β-sheet^41^,^42^. This is well in accordance with previous studies where the amide I’ maximum for TTR in D_2_O was determined to 1630 cm^−1^ 43. The choice of solvent affected the peak maxima shifting the value to a slightly higher wavenumber when using H_2_O instead of D_2_O. Minor peaks associated with the presence of α-helix content (1653 cm^−1^), turns and anti-parallel β-strands within the sheet (1668 and 1686 cm^-1^) were also observed^41^,^42^, in accordance with the crystal structure of tetrameric TTR (Fig. 7D). The protofibrils revealed a spectrum with a maximum around 1622 cm^−1^ associated to the formation of a stacked β-sheet in the protofibril^44^. Minor peaks associated to disordered structures (~1645 cm^−1^) and α-helix content (~1655 cm^−1^) were also registered 41. A strong peak at 1691 cm^−1^, along with the main peak around 1622 cm^−1^, suggest that the protofibrils are constituted mainly by repetitive non-native anti-parallel stacked β-sheets. It should, however, be noticed that a distinction between parallel and anti-parallel β-sheets is difficult using FTIR^45^, notwithstanding the 1691 cm^−1^ peak strengthen the conclusion of anti-parallel β-strands comprising the protofibril core^46^.

## Discussion

The mechanistic details of *in vitro* TTR fibrillation have been widely studied and increasingly convincing models are emerging^11^,^19^,^22^,^26^,^47^. However, these models disagree and lack structural details both on on-pathway species and on fibril structure. In the present work we investigate the fibrillation process of 5 mg/ml TTR in 50 mM acetic acid with 100 mM NaCl (pH 3.0) at 4 °C. The combination of an in-depth SAXS study of TTR fibrillation process and HXMS analysis of the formed protofibrils provides a description of the species present throughout the process, mechanistic details about the principles of protofibril formation and details about protofibril structural dynamics. Together, the data provide the means of proposing a novel mechanism of TTR fibrillation under the conditions investigated. Most importantly, our studies direct attention to a considerably unfolded monomeric TTR species, continuously present in equilibrium with the native tetramer and the protofibril throughout the fibrillation process. These observations have a significant impact on the conceptual understanding of the TTR protofibril structure and formation mechanism.

During the very early phases of TTR fibrillation, ThT fluorescence increases continuously but very slowly. In this apparent lag-phase, first ill-defined, later early protofibrils appear (Fig. 2). The onset of fibrillation is independent of concentration under the experimental conditions investigated (Fig. 1). The aggregates formed under the conditions investigated here do not grow to μm length scales, but reach a maximal dimension of less than 100 nm (Figs 2, 4 and 6). Interestingly, upon maturation, these protofibril species homogenize and reach a globally shorter maximal dimension (Fig. 4B, Supporting Table S1). Our HXMS analysis reveals an unexpectedly high solvent exposure for mature protofibrils (Fig. 7) compared to other studies of TTR amyloid fibrils^27^. In fact, the only protected segment is the F-strand (E92-F95). This can in principle be interpreted such, that the only densely structured part of the protofibrils is this short segment, which hence would constitute the protofibril core. The remaining parts of TTR would then be in a loose and solvent exposed state. While this short F-strand segment indeed is identified as the most amyloidogenic segment according to the Waltz prediction algorithm^48^, and several models suggest the F-strand to be hidden in the fibril core^22^,^49^, the notion of an almost completely unstructured and loosely connected protofibril structure is highly implausible. Our CD and FTIR data on the other hand show that the protofibril state includes a high proportion of β-pleated structure, which is significantly different than the native state but not unstructured. These data hence contradict the immediate HXMS-based suggestion of a very loose and solvent exposed protofibril structure. Even if the β-pleated structure is evident from the FTIR and CD data, it was evident that a significant proportion of the peptide chain is in an unfolded state, and that residual α-helix may be present (Fig. 8).

**Figure 9:**
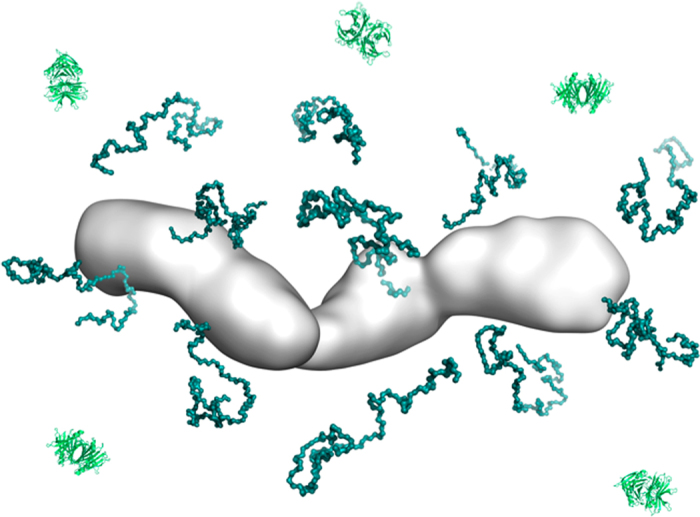
Illustration of the dynamic nature of TTR protofibrils. Considerably unfolded TTR monomers (teal structures) are omnipresent during TTR fibrillation and exchange between the solution state and the protofibril state. A protofibril is shown in white surface presentation and a few tetrameric native TTR structures are shown in cartoon presentation in light green.

These observations also do not correspond well with the general conception that the fibril state is a tightly packed and very stable structural state consisting primarily of extended β-sandwiches in the fibril core. In the light of our SAXS data, however, our concerted observations reveal a novel aspect of the protofibril formation principles and structural state, as summarized in Fig. 9.

*In vivo*, the amyloid fibril formation process of WT TTR initiates from the folded tetramer^50^ after dissociation. Likewise, under the *in vitro* conditions examined, tetrameric TTR is the most prevalent species present prior to fibrillation initiation (Fig. 3), remaining present throughout the elongation process (Fig. 4B). However, a novel important observation is the continuous equilibrium between a non-native monomer and the native tetramer both prior to initiation and during fibrillation (Figs 3, 5 and 9). Such an equilibrium has previously been observed under other experimental conditions for TTR^51^. In that previous study, however, it was demonstrated that protein fibrillation required a structurally well-defined folding intermediate, which was concentration–dependent, and became non-amyloidogenic when further unfolded (below pH 3). Other previous studies also suggest the presence of a near-native species during fibrillation^52^,^53^,^54^. In the above works, the overall stability of the native state TTR at pH 4.5 and pH 5.75 for WT TTR and a molten globule state at pH 2.0 (in HCl) is compared by NMR, including comparison to known disease-associated mutants at pH 7.0, revealing a stable core, while hinge regions of increased flexibility is observed. The authors hence suggest that such mobility may play a role in the onset of fibrillation. We do not observe the presence of such a partially folded molten globule state in our samples. While a lack of observation is no direct proof of absence of low volume fractions of a molten globule state, we can firmly conclude that we can fit our data without such a species. In our study, in contrast, we show that the fibrillation is concentration independent within the range investigated. Moreover, the intermediate monomer is considerably unfolded and present in equilibrium with native tetramer (Fig. 3), and the monomer remains in equilibrium with the growing and matured protofibril. Hence, the SAXS data that we have collected throughout the fibrillation process can be fitted well (Fig. 5) by a combination of native tetramers, protofibrils, and an inclusion of approximately 15% volume fraction of significantly unfolded TTR (the latter observed experimentally in 11% acetic acid (Fig. 3)).

The observation that such a considerably unfolded monomer is omnipresent in solution offers the key to understanding the intriguing biophysical observations. When analysing the protofibril samples by FTIR, CD and HXMS, the monomeric species is also present in a volume fraction of approximately 15%. Hence, when collecting data from the protofibril state, the data will include a summed signal from both the in-fibril and unfolded monomeric state. This means, that the large degree of unfolded structure and the strong exposure to solvent reflects not directly the in-fibril protomers, but rather the combined state, where the monomers are partially present in solution, partially present in the protofibril. The overall maturation and homogenisation of the length of protofibrils, is hence a reflection of a significant exchange of protomers from the protofibrils samples. The protofibrils are thus of a highly dynamic nature, where the protomers constantly interchange between being embedded in the fibril, and being present in the solvent in a very unfolded state (Fig. 9). This means, that even when trying to isolate the protofibril state (e.g. by centrifugation) an equilibrium with the soluble unfolded state will immediately re-form, thus the biophysical data will always reflect the combined state. This is much in contrast to the general notion that the fibril structure is a highly stable and near-irreversible structural state. Our conclusion is thus surprising and has notable consequences.

It is notable that the initial brief lag-phase in TTR fibrillation does produce initial aggregates, both ill-defined and protofibrils. It is thus possible that the TTR lag-phase under the current experimental conditions, rather than being a nucleation-dependent phase, reflects competing pathways between fibril and non-fibril aggregate formation. Our continued analysis reveals that the pre-initiation equilibrium between non-native monomer and native tetramer persists throughout fibrillation, having a near-constant proportion between structured (protofibrillar + native tetramer) and unfolded monomers. We can thus propose that the considerably unfolded monomer is present in the maximally allowed concentration, throughout the fibrillation process, and that the solubility of this species determines the initial elongation rate.

It is interesting to consider this suggestion in the light of a previous study on a monomeric mutant of TTR (M-TTR)^11^. Here, it was shown that the onset of fibrillation was nucleation independent, and followed what was best described as a downhill polymerisation pathway. If an unfolded monomer, conceptually comparable to the monomer observed in the present study, would have been present in equilibrium with M-TTR, it is possible that onset of fibrillation would depend strictly on the concentration of this monomer. In Hurshman *et al.*^11^, the experimental conditions were not comparable to the present study, thus comparison should be made with care, but our suggestion provides a new principle of interpretation of these data. In the M-TTR study, a clear concentration dependency was revealed. It is possible that equilibrium between the folded and unfolded M-TTR is concentration dependent, which thus is not the case within the tested experimental range for native tetrameric TTR, as revealed in our study.

Protofibrils form almost immediately, already during the brief lag-phase, and continue forming during an approximate 10 hours rapid exponential elongation period, which is fed by depletion of native tetramers, which dissociate and immediately unfolds. This period is followed by a slower period of conversion into mature protofibrils. The end of the immediate elongation period and transition to the maturation phase must depend on the availability of new non-native monomers. This state thus represents an intermediate pseudo-equilibrium state. The state is only in pseudo-equilibrium since protofibrils interchange protomers with the solution state, and thus a different and slower maturation process follows.

At no point do we detect dissociated native species (folded monomers or dimers) or spherical aggregates. The latter, observed by TEM, are probably induced by surface effects, which we do not have in SAXS. Neither are any other oligomers observed downhill from dissociation, such as multimers of dimers or re-arranged tetramers. Oligomer accumulation has been observed for all fibrillation processes previously studied in depth by SAXS, including insulin, glucagon and α-synuclein fibrillation^34^,^35^,^36^. The lack of an oligomeric building block shows that TTR follows a fundamentally different fibrillation process. Previous studies using size exclusion chromatography showed that dimeric or hexameric intermediates lead to large fibrillar aggregates^6^. Another study by us has shown that cytotoxic oligomeric TTR in the range of 20-30-mers forms rapidly prior to fibrils, but in the absence of a lag-phase^7^. However, these TTR intermediates were observed at different experimental conditions.

Several fibril morphologies have been observed for TTR^15^,^30^,^31^,^33^,^47^,^55^,^56^. These morphologies depend on the fibrillation conditions and potential mutations in the TTR sequence^7^,^8^,^11^,^29^,^32^. Certain interlinked TTR dimers form protofibrils^22^ while other stabilised dimers do not form fibrils^57^. We can hence not exclude that both monomeric and oligomeric self-assembly mechanisms could exist for TTR under different experimental conditions. Hence, the formation of considerably unfolded monomeric TTR which is clearly revealed under the current experimental conditions, is not necessarily an obligate step in the formation of all types of TTR fibrils^22^.

We observed a slow, almost steady phase in the ThT signal after approximately 10 hours, but our SAXS data reveal that an overall rearrangement of protofibrils takes place, going from a rather heterogeneous protofibril population, including protofibrils of varying lengths, to a more homogeneous population. This observation, in itself, suggests exchange of protomers between the protofibrils: continued exchange of non-native monomers from the protofibril state to the solution state, and back into the protofibril state will, over time, lead to protofibril structures of homogeneous length. We can immediately conclude that this exchange is slow, compared to the initial assembly rates. This is also directly evident from our SAXS data, since the second crossover point in the SAXS data curves only appears at late timepoints, when the elongation rate slows down (Fig. 4). Our HXMS data further reveal that the interchanging species is greatly exposed to solvent, and the SAXS data can be fitted, by including the scattering curve from a highly unfolded monomeric species. In concert, this suggests that there is a high on-off rate from the protofibrils, where non-native monomers are released from the protofibril structure, thereby causing the high average solvent exposure rates observed by HXMS (Figs 7 and 9). The protofibril structure is hence not a single structural state. The total population of TTR is distributed between the protofibril and solution state, and one state is not independent on the other. The TTR protofibrils are thus best described as a multistructural state.

Although our observations have been made under a specific set of experimental conditions, it is worth considering whether such on-off interchange exists under other conditions as well. Importantly, such a condition could offer an alternative explanation for heterogeneous catalysis^58^ (a.k.a. the auto-catalytic effect) observed in most fibrillating systems. In simple terms, the auto-catalytic effect means that the presence of fibrils stimulates the formation of new fibrils. This is suggested to be caused by fibril surface characteristics, via e.g. template-guided aggregation or via simpler causative mechanisms such as fibril branching and breaking. Here, however, we reveal that fibrillation-prone protomers are interchanging between fibril and solvent state. This has the direct consequence that the concentration of unfolded and fibrillation-prone monomers is higher near the fibril surface (Fig. 9), and this inevitably will lead to the formation of new fibrils. Our observation thus provides a new model for secondary nucleation.

Our finding that TTR protofibrils are in constant interchange with a considerably unfolded monomer also suggests that the protofibril state is degradation prone. The HXMS data reveal a particular disordered structure within the first 50 residues of TTR protofibrils (i.e. the fraction of protofibrils in the solution state), which is consistent with the findings of fragmented TTR within the majority of *in vivo* deposits^59^,^60^. Although the currently reported observations are made *in vitro* under non-physiological conditions, it is interesting to speculate whether *in vivo* conditions would still accommodate for the presence of such a monomeric unfolded species. If *in vivo* fibrils also interchange with a solution state unfolded protomer, this could explain the observed disease-related fragmentation. This also translates to different clinical outcomes for patients carrying the common V30M mutation^61^. Such fibril heterogeneity is reminiscent of amyloid strains conceptualised for prions.

Our data strongly support the recently developed concept of a small-molecule kinetic stabilizer as treatment against TTR-mediated FAP^10^. This class of compounds has been developed specifically for stabilizing the native tetrameric structure in order to avoid the formation of any non-native monomeric species. Indeed, the stabilization of the tetrameric structure would assumingly significantly diminish the presence of the considerably unfolded monomer, which is central for both elongation and maturation of protofibrils in this study.

Here, we describe the presence of a non-native monomeric TTR state, which only exists in equilibrium between the native and protofibril state. This molecule can neither be isolated, nor can it be described by a single structural state and traditional structure based drug development strategies are accordingly compromised. However, the presence of the monomeric species in rapid exchange with the larger oligomeric states will definitely significantly influence the physiological impact of such a structural state, hence must be taken into account in therapeutic approaches. The concept of drug development against rare or unstable species and structural ensembles must thus follow other principles than those based on detailed structural information from steady state protein structures.

Based on our concerted findings we propose the following model for the assembly of TTR protofibrils at the given conditions: fibrillation initially proceeds from a solution with a dynamic equilibrium between a small fraction of considerably unfolded monomers and natively folded tetramer. This monomer is likely to have a fibrillation prone conformation, which can either misassemble and elongate protofibrils under denaturing conditions or under ambient conditions re-associate into the native tetramer. We observe no novel oligomeric building block accumulating at any time during fibrillation. Rather, we advocate for an elongation model with a monomeric building block. This same monomeric state interchanges between the protofibril and soluble state, thereby causing maturation of the final fibril structure. The maturation phase is considerably slower than the elongation phase.

Our observations provide a novel suggestion for the autocatalytic effect causing exponential growth during elongation. Also, if such interchanging species, which we have observed *in vitro* under acidic conditions should be present *in vivo*, this would certainly play a role that has not previously been considered when debating potential membrane modulation and amyloid-associated cytotoxicity. Our findings stimulate to novel strategies in future drug development against TTR-associated amyloidoses.

## Methods

### Materials

ThT was purchased from Sigma Aldrich Chemie GMbH (Schnelldorf, Germany). A molar extinction coefficient of 36,000 M^−1^ cm^−1^ at 412 nm was used to determine the dye concentration. 1 mM ThT in milli-Q water was prepared, filtered (Millex®GP Filter unit 0.22 μm PES Membrane), and stored protected from light at 4 °C. D_2_O (99.9 atom % D) was obtained from Merck (Darmstadt, Germany). All other chemicals were of analytical grade.

### Protein production and purification

WT TTR expressed in BL21 (DE3) cells transformed with the pmm Hα:WT TTR plasmid was isolated and purified as previously described in Groenning *et al.* 2011^32^. The protein was dialysed against milli-Q water at 4 °C using a dialysis tube (Spectra/Por®, Spectrum Laboratories) with a volume/length of 1 ml/cm and a molecular weight cut-off (MWCO) of 3.5 or 12–14 kDa and concentrated to 5.2 mg/ml using spin-filters (Centriprep, Millipore) with a MWCO of 10 kDa at 4 °C. For concentration determination a molar extinction coefficient of 18,289 M^−1^ cm^−1^ at 280 nm was applied.

### Fibrillation procedure

Prior to the initiation of TTR fibrillation process, the sample was centrifuged for 60 s at 13,400 rpm (Denville 260D, Denville Scientific Inc). The experimental conditions for the fibrillation reaction were optimized such as to allow for SAXS data collection from adequately concentrated protein samples, within a time-window of maximally 24 hours^38^. The fibrillation of 5 mg/ml TTR was induced by adding a stock solution of 1 M acetic acid, 2 M NaCl to a final concentration of 50 mM acetic acid and 100 mM NaCl (final pH 3.0). The sample was mixed using a Vibromix (Practical Science, Nederlands) for 5 s placed in a waterbath at 4 °C. Aliquots were taken as needed for further analysis. Mature protofibrils were obtained by incubation at these conditions for 1 month.

### Thioflavin T fluorescence

*Ex situ* ThT measurements^62^ were performed during fibrillation by transferring a 10 μl aliquot of the TTR solution to a cuvette with 1 ml 20 μM ThT in 50 mM phosphate buffer 100 mM NaCl (pH 7.5). For further details, please refer to Supporting Information. The fluorescence intensity was plotted as a function of time and was fitted with a sigmoidal curve (logistic 3 parameter) in Sigma Plot to guide the eye. The initial phase fibrillation kinetics at different concentrations (5, 1, 0.5, 0.25, and 0.1 mg/ml TTR) were also examined using ThT fluorescence (average ± std. dev. n = 1 or 2). The time of the apparent initial lag-phase was estimated as the first time point where the fluorescence intensity significantly exceeds the rest (exceeds the background with 40 arbitrary fluorescent intensity units).

### Transmission Electron Microscopy

Aliquots were taken at different time points throughout fibrillation. In addition, mature fibrils were formed from unfolded TTR (10 mM HCl, no salt, 4 °C) in fibril promoting conditions: 1 mg/ml TTR, 10 mM HCl, 100 mM NaCl at 22 °C for >one month. After dilution (1:100 in milli-Q water), 5 μl were placed on a carbon coated copper grid for 2 min. The remaining drop was blotted dry using a filter paper. The grid was then rinsed by applying a 5 μl water droplet. The samples were stained using 2% uranyl acetate for 20 s. A Jeol JEM1230 transmission electron microscope operating at 100 kV was used for obtaining micrographs at 100,000-fold magnification.

### Small Angle X-ray scattering data

Data were collected at beamline X33 at the European Molecular Biology Laboratory, Hamburg on DORIS III (DESY)^63^ at a wavelength of 1.5 Å. A MAR345 Image Plate detector was used in the momentum transfer range 0.07307 < q < 4.945 nm^−1^ (q = 4π sin θ/λ, θ is half the scattering angle and resolution = 2π/q). Exposure time for each sample was two minutes. The measurements were performed at 4 °C. Selected TTR samples exposed to repeated exposure showed no detectable radiation damage. Data were corrected for detector response and scaled to the intensity of the X-ray beam, protein concentration, and radially averaged following standard procedures of the beamline. SAXS data were collected from samples extracted from the fibrillating solutes. The incubation happened in two eppendorf tubes each loaded with 1.5 ml of TTR solute at 4 °C. 50 μl samples were withdrawn at appropriate time points (Tube 1: 3½, 25, and 45 minutes, 1 h, 1 h 45 min, 3 h 10 min, 7, 9, 12½, 14½, and 21½ h and Tube 2: 10 and 40 minutes, 2, 4, 6, 9, 11, and 18 h). Furthermore, SAXS data were collected on the pre-initiation conditions of 5.2 mg/ml TTR in milli-Q water and on 6.6 mg/ml TTR in 11% acetic acid (unfolded monomer). Data analysis was performed using the software suite ATSAS 2.3^64^, employing PRIMUS for data reduction^65^. For further details, cf. Supporting Information. P(r) was estimated using the indirect Fourier transformation program GNOM^66^, yielding D_max_, average R_g_ and estimated forward scattering values. R_g_ and MW were plotted as a function of time and fitted using a sigmoidal function (logistic, 4 parameter) in Sigma Plot. Kratky plots (I(q)* q^2^ versus q) were generated using the program PRIMUS^65^. SVD was performed using the program SVDPLOT^65^ to estimate the number of components significantly contributing to the scattering during the fibrillation process (21½ hours). Data in the q-range from 0.230 to 4.95 nm^−1^ were applied.

### Theoretical scattering curves and rigid body fitting

Using the program CRYSOL^67^, theoretical scattering curves were calculated using 25 harmonics, 200 or 256 points and a maximum q-value of 4.95 nm^−1^. The theoretical scattering from a TTR tetramer, dimer and monomer were calculated based on two crystal structures (1TTA.pdb and 3CBR.pdb). The latter crystal structure is crystallized at pH 3.5, referred to as amyloidogenic pH^68^. Missing residues in the N- and C-terminal of TTR (residues 1–10 and 124–127) and these residues were added by alignment with 1TTA. Missing loop residues 37–38, 76–85 and 99–102 were not possible to adapt from 1TTA.pdb due to imperfect alignment. Furthermore, spheres with diameters of 15, 20, 30 and 40 nm were generated using the program DAMMIN53^69^ and the theoretical scattering curves were calculated. Rigid body refinement was performed for 1TTA.pdb AB dimers with the contact condition file (1 20 21 2 113 114) and a contact distance of 15 Å using SASREF^70^.

### Deconvolution of SAXS data

The program OLIGOMER^65^ was employed to fit the experimental data curves with linear combinations of the scattering from individual components, yielding their volume fractions. In the cases where both experimental and theoretical data curves were applied, they were scaled and joined into an output file using the program JOIC4qw in the ATSAS package. Data in the q-range from 0.230 to 4.95 nm^−1^ were applied. The quality of the fit was evaluated using the discrepancy (χ^2^) value between the experimental data and the fitted curve and by visual inspection of the fit.

### Ab initio modelling of SAXS data

An *ab initio* structure of the fibril was obtained using the program DAMMIN53^69^. A simulated annealing protocol was used to build a compact bead model of uniform scattering length density, which minimises the discrepancy between the experimental and calculated curves at low resolution (q < 0.72 nm^−1^). Standard penalties (looseness penalty weight = 0.002, disconnectivity penalty weight = 0.002 and peripheral penalty weight = 0.3), 20 spherical harmonics, annealing schedule factor of 0.95 and no symmetry constraints were applied. The initial search volume for the fibril was estimated based on D_max_ and shape of the P(r). A cylindrical search volume with a length of 80 nm, a radius of 20 nm and 9.118 dummy atoms were used. 20 individual jobs were computed on a Linux cluster and averaged using the program DAMAVER^71^. A final averaged and filtered model with an excluded volume in accordance with the individual models was calculated. The NSD of 20 models was calculated. The excluded volume of the hydrated particle was obtained from the individual *ab initio* models. The MW (kDa) of the model is approximately estimated as 1/1.700 of the excluded hydrated volume (in Å^3^)^72^.

### Amide hydrogen (1H/2H) exchange monitored by mass spectrometry (HXMS)

Samples were prepared from a concentrated stock solution of TTR in the native or protofibril state, without further handling such as centrifugation or chromatography. Isotopic exchange was initiated by diluting 6 μl 200–400 μM of each protein sample in 250 μl 0.1% (v/v) trifluoroacetic acid (TFA) in D_2_O buffer (pD 2.5 uncorrected value). The exchange was carried out at room temperature for 1 min, 5 min, 15 min, 60 min, 3 h, 9 h, 24 h, 3 days and 7 days. The samples were snap-frozen in liquid nitrogen. To determine the degree of back-exchange in the system, fully deuterated protein was prepared by incubation of the samples in 9 M deuterated urea at pH 7.4 overnight at 25 °C. The Liquid Chromatography (LC) setup is based on the system described previously^73^, with the following modifications: aqueous solution for desalting was delivered by a Pharmacia LKB High-Performance Liquid Chromatography (HPLC) pump 2248, while an organic gradient for elution of peptides/proteins was delivered by a Brownlee Labs Microgradient system (Applied Biosystem). Samples were loaded onto a column packed with immobilised pepsin (Pierce, Rockford, Illinois, USA) and digested for 15 s. The peptic peptides were desalted for 2 min and eluted with a 9 min gradient (7 min 12% to 40% acetonitrile, 0.05% TFA and two additional minutes 40% to 80% acetonitrile, 0.05% TFA). The LC system was coupled to a QToF I (Waters/Micromass) mass spectrometer. Spray voltage was 3.5 kV, cone voltage 40 V, and ion source block temperature 120 °C with a desolvation gas flow of 500 l/h at 200 °C and nebulising gas flow of 20 l/h at room temperature. The HXMS data were analysed by HX-Express^74^. To identify peptic peptides, non-deuterated protein was digested and desalted as described above. The peptic peptides were eluted and collected for nanoflow-LC-MS/MS analysis, which was carried out with an Ultimate (LC Packings) nanoLC coupled to the QToF I. Data-dependent acquisition was employed to obtain MS/MS spectra of the peptic peptides. The peptides were identified by the Mascot^75^ MS/MS ion search engine using an in-house database. The H56-E63 segment was calculated by subtracting the deuterium incorporation of V28-E63 and V28-L55 peptic peptides. The E92-F95 segment was calculated by subtraction of the T96-A110 and E92-A110 peptic peptides.

### Fourier Transform Infrared Spectroscopy

FTIR spectra were recorded at room temperature using a BOMEM MB104 FTIR spectrophotometer (Québec, Canada). For each spectrum, 256 scans were collected in the range 4000–400 cm^−1^ with a resolution of 4 cm^−1^. The samples were prepared as hydrated thin films, having a path length of 6 μm, between two CaF_2_ discs. The sample chamber was purged with N_2_. All spectra were corrected for background signal prior to further data processing. First, a spectrum of the appropriate buffer was subtracted to achieve a straight baseline from 1800–2600 cm^−1^. Secondly, a water vapour spectrum was subtracted to correct for noise in the amide I band region 1700–1500 cm^−1^ and to smoothen the baseline. Second-derivative spectra were obtained with Savitsky-Golay derivative function with an 11 data point window. Area-normalisation to an area of one was conducted on the region 1705–1595 cm^-1^ for comparison reasons. All data processing was performed with the GRAMS/AI software, version 7.0 (Thermo Galactic).

### Circular Dichroism Spectroscopy

CD spectra were recorded at 4 °C using a Chirascan CD spectrometer (Applied Photophysics) and a 0.1 mm path length quartz cell (Hellma). Briefly, native TTR and mature protofibrils were diluted 4x using distilled water, making sure the original pH (7.5 and 3, respectively) was maintained. CD scans were acquired from 185 to 280 nm, with a 1 nm bandwidth and a 1 nm step resolution. Ten scans were averaged for each sample and background absorbance was subtracted. All spectra were normalized to the mean residue ellipticity (θ_MRE_) [deg cm^2^/dmol] using the equation θ(λ)_MRE_ = θ(λ)_mdeg_/*cnd*, where θ(λ)_mdeg_ is the recorded spectra in millidegrees, *d* is the path length of the cell in centimeters, *n* is the number of amino acid residues, and *c* is the molar concentration of the protein.

The secondary structures in the native state of TTR were obtained by judging from analysis of high resolution (<1.5 Ǻ) crystal structures of TTR (1F41.pdb, 4FI8.pdb and 3M1O.pdb using the DSSP algorithm^76^. The CD spectra was deconvoluted into secondary structure components using the CDSSTR algorithm^77^ from Dichroweb^78^,^79^.

## Additional Information

**How to cite this article**: Groenning, M. *et al.* Considerably Unfolded Transthyretin Monomers Preceed and Exchange with Dynamically Structured Amyloid Protofibrils. *Sci. Rep.* **5**, 11443; doi: 10.1038/srep11443 (2015).

## Supplementary Material

Supplementary Information
