# Supplementary material for: Considerably Unfolded Transthyretin Monomers Preceed and Exchange with Dynamically Structured Amyloid Protofibrils
Source: Sci Rep. 2015 Jun 25;5:11443. doi: 10.1038/srep11443 (PMC4480009; doi:10.1038/srep11443)
Supplement: Supplementary Information [file srep11443-s1.doc]

**Supporting Information**

Considerably Unfolded Transthyretin Monomers Preceed and Exchange with Dynamically Structured Amyloid Protofibrils

*Minna Groenning^a,b^, Raul I. Campos^b^, Daniel Hirschberg^b^, Per Hammarström^b*^ and Bente Vestergaard^a*^*

^a^Department of Pharmacy and Department of Drug Design and Pharmacology, University of Copenhagen, Copenhagen, Denmark

^b^IFM – Department of Chemistry, Linköping University, Linköping, Sweden

** To whom correspondence should be addressed*

**Supporting Information**

**Thioflavin T fluorescence measurements, details**

The fluorescence was measured using a Hitachi F-4500 spectrofluorometer with a 150 W Xenon lamp. An excitation wavelength of 440 nm was used and an emission wavelength scan was performed from 455 to 530 nm. The scan speed was 240 nm/min, while the PMT voltage was 950 V. The excitation and emission slits were 5 nm and all measurements were carried out at 4 °C. The fluorescence intensity for each sample was compared at their peak maximum (in the range 484 to 491 nm) after background subtraction.

**SAXS data reduction, details**

In the SAXS data curves visualised, four data points were binned. However, the data analysis was done on the full unbinned data curve, unless otherwise stated. R_g_ and the average MW of solutes were estimated from the extrapolated relative forward scattering, I(0) using the Guinier estimation and using bovine serum albumin at 6.7 mg/ml as standard. The Guinier approximation was valid the first two hours, after which the presence of very large scatterers in the solution compromised the estimates. Later estimates presented were obtained using the AutoRg function in PRIMUS. These should be regarded as rough estimates and are only representative for samples until six hours, after which the sample was heavily fibrillated.

**Table S1** Guinier and GNOM estimation of R_g_, D_max_ and MW during the TTR fibrillation process.

| **Time** | **Guinier**  **R_g_ (nm) MW (kDa)** | | **GNOM**  **R_g_ (nm) D_max_ (nm)** | |
| --- | --- | --- | --- | --- |
| Pre-initiation | 2.6 | 45 | 2.5 | 7.2 |
| 3½ min | 3.0 | 41 | 3.1 | 12.5 |
| 10 min | 3.1 | 39 | 3.5 | 15 |
| 25 min | 3.1 | 46 | 3.3 | 13.5 |
| 40 min | 3.1* | 54 | 3.5 | 14 |
| 45 min | 3.1 | 48 | 3.5 | 14 |
| 1 h | 3.4 | 55 | 4.1 | 17.5 |
| 1 h 45 min | 4.3* | 65 | 5.1 | 19.5 |
| 2 h | 4.3* | 74 | 5.2 | 21 |
| 3 h 10 min | 4.9* | 63 | 6.5 | 28 |
| 4 h | 6.4* | 135 | 8.6 | 33 |
| 6 h | 13.3* | 367 | 15.5 | 56 |
| 7 h | 13.2* | 377 | 15.3 | 57 |
| 9 h | 14.0* | 526 | 16.5 | 59 |
| 9 h | 15.1* | 617 | 17.4 | 61 |
| 11 h | 15.5* | 702 | 17.4 | 61 |
| 12½ h | 15.5* | 679 | 18.0 | 66 |
| 14½ h (outlayer) | 13.3* | 462 | 14.3 | 51 |
| 18 h | 17.0* | 981 | 20.3 | 71 |
| 21½ h | 17.5* | 1075 | 20.7 | 72 |
| Mature protofibrils | 18.5* | 832 | 21.1 | 70.5 |

*Some larger aggregated species present, as evaluated by AutoRg function in PRIMUS

**Table S2** OLIGOMER analysis of the composition of TTR in the pre-initial and initial phase*.

| **Time** | **Pdb-file** | **Volume fraction**  Unfolded monomer monomer dimer tetramer | **χ** |
| --- | --- | --- | --- |
| Pre-initiation | 1TTA.pdb^a^  1TTA.pdb^a^  3CBR.pdb^b^  3CBR.pdb^b^ | 15% 0% 0% 85%  Not included 1% 0% 99%  13% 0% 0% 87%  Not included 0% 0% 100%  5% 0% 0% 95%  Not included 0% 0% 100%  3% 0% 0% 97%  Not included 0% 0% 100%  18% 0% 0% 82%  Not included 0% 0% 100%  18% 0% 0% 82%  Not included 0% 0% 100% | 1.89  2.20  2.85  3.01  4.07  4.31  5.35  5.58  4.92  5.60  6.28  6.83 |
| 3½ min | 1TTA.pdb^a^  1TTA.pdb^a^  3CBR.pdb^b^  3CBR.pdb^b^ |
| 10 min | 1TTA.pdb^a^  1TTA.pdb^a^  3CBR.pdb^b^  3CBR.pdb^b^ |

*Unfolded monomer, native monomer (monomer), native dimer (dimer) and native tetramer (tetramer) are applied in the analysis. ^a^ Native structure formed at pH 3. ^b^ Native-like structure formed at pH 3.5. The structure of 3CBR is deficient and is therefore modified with N- and C-terminals and segments in the B-chain (37-38 + 76-85 + 99-102) from the 1TTA.pdb structure.

**Table S3** Percent (%) distribution of the structural element fractions for native and protofibrillar TTR obtained by deconvolution of CD spectra using CDSSTR ^1^ algorithm and reference database 4 ^2^, available on Dichroweb ^3^.

| **TTR** | **α-helix β-sheet Remainder^a^ NRMSD^b^** |
| --- | --- |
| Native | 6 45 49 0.006  13 33 54 0.011 |
| Protofibrils |

^a^ Turns + unordered structures. ^b^ The NRMSD parameter represents the normalized root mean square deviance.

**
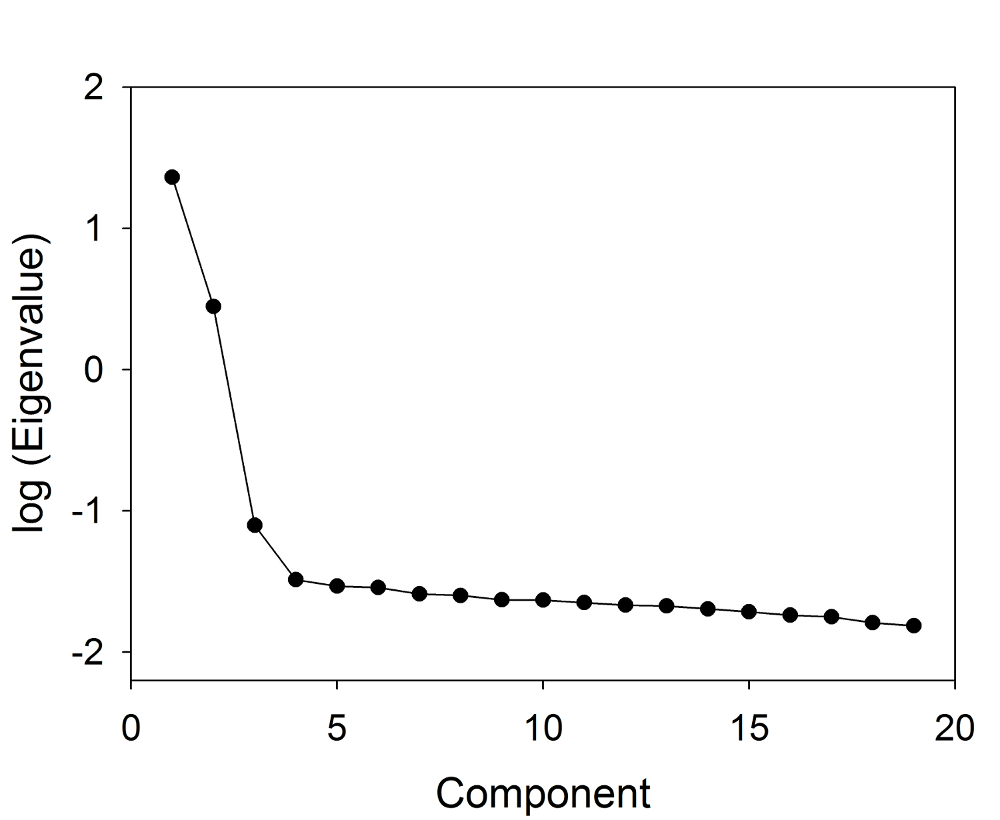
**

**Figure S1** **Singular Value Decomposition of the scattering data set collected during the fibrillation process.** Logarithm to the eigenvalues is shown as a function of the ordinal number of the component. The first three components significantly exceed the remaining component corresponding to three principal species being present: Two major and one minor species.

**
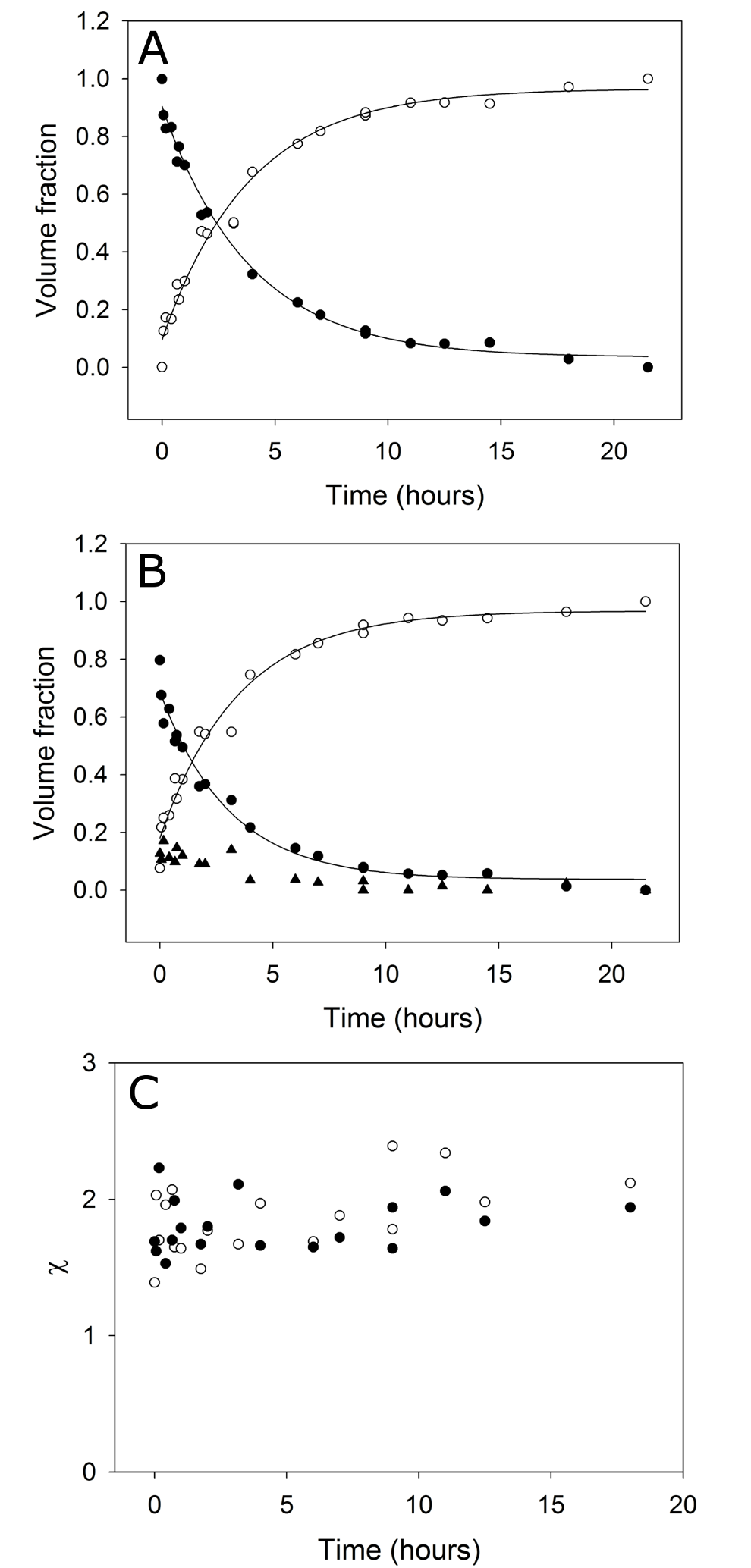
**

**Figure S2** **Alternative decomposition of the experimental scattering curves.**

Decomposition of experimental scattering curves using two complementary approaches resulting in the same conclusion. In **(A)** the experimental data are fitted with a linear combination of the experimental scattering curves for the pre-initiation tetramer (solid spheres) and the protofibrils after 21½ hours of incubation (open spheres) weighted by their volume fractions. Volume fractions are shown as a function of time. In **(B)**, the experimental curve for the pre-initiation state is replaced by a combination of the experimental scattering curve of the unfolded monomer and a theoretical scattering curve for the tetramer. This is possible, because we have already shown that the pre-initiation state is a combination of monomer and tetramer. However, when fitting the experimental curves for the fibrillation reaction, we allow the volume fractions of both fibril, monomer and tetramer to vary. The resulting volume fractions are shown for the unfolded monomer (solid triangles), the the crystallographic (1TTA) tetramer (solid spheres) and the protofibrils after 21½ hours of incubation (open spheres). To guide the eye the volume fractions are fitted by a single exponential decay function (3 parameters) (solid spheres) and a single exponential rise to maximum (3 parameters) (open spheres). **(C)** A comparison of the fit for the two complementary approaches. The discrepancy between the fit of the decomposed scattering curves and the experimental data is shown as a function of time. The decomposed scattering curves consist of either the pre-initiation tetramer and protofibril after 21½ hours (open spheres) or unfolded monomer, 1TTA tetramer, and protofibril after 21½ hours (solid spheres).

**
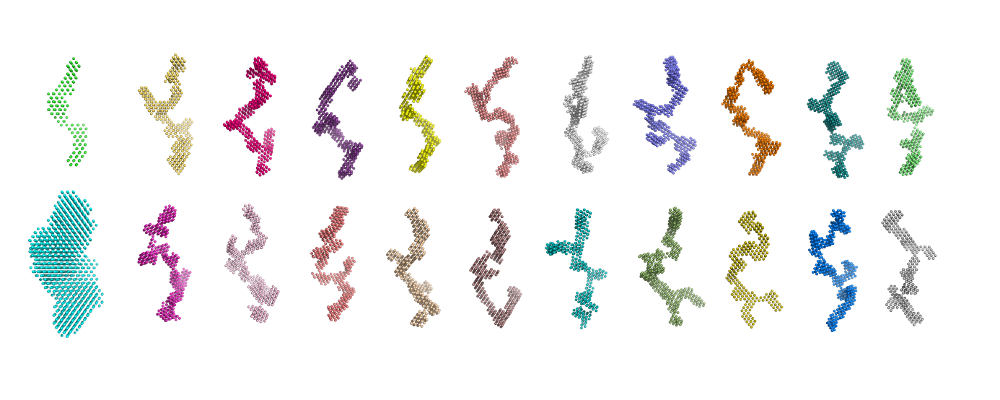
**

**Figure S3 Ab initio models of the TTR protofibril.** Visualisation of the filtered (upper, first from left) and averaged (lower, first from left) model as well as the twenty individual models of the mature protofibril. Some individual models had a tendency to branch into two robes and the twisted fibril structure often had small arms.

Supporting Reference List

1. Sreerama,N. & Woody,R.W. Estimation of protein secondary structure from circular dichroism spectra: Comparison of CONTIN, SELCON, and CDSSTR methods with an expanded reference set. *Anal. Biochem.* **287**, 252-260 (2000).

2. Janes,R.W. Reference Datasets Circular Dichroism and Synchrotron Radiation Circular Dichroism Spectroscopy of Proteins in *Modern Techniques in Circular Dichroism and Synchrotron Radiation Circular Dichroism Spectroscopy* (eds. Wallace B.A. & Janes R.W.) (IOS Press 2009, 2008).

3. Whitmore,L. & Wallace,B.A. Protein secondary structure analyses from circular dichroism spectroscopy: Methods and reference databases. *Biopolymers* **89**, 392-400 (2008).
